# Hepatorenal Syndrome

**DOI:** 10.4236/ijcm.2014.53018

**Published:** 2014-01

**Authors:** Tyree H. Kiser

**Affiliations:** Department of Clinical Pharmacy, University of Colorado Skaggs School of Pharmacy and Pharmaceutical Sciences, Anschutz Medical Campus, Aurora, USA.

**Keywords:** Hepatorenal Disorder, Cirrhosis, Portal Hypertension, Transplantation, Vasopressors

## Abstract

Hepatorenal syndrome (HRS) is the most serious hepatorenal disorder and one of the most difficult to treat. To date, the best treatment options are those that reverse the mechanisms underlying HRS: portal hypertension, splanchnic vasodilation, and/or renal vasoconstriction. Therefore, liver transplantation is the preferred definitive treatment option. The role of other therapies is predominantly to prolong survival sufficiently to allow patients to undergo transplantation. Terlipressin with the addition of adjunctive albumin volume expansion is the preferred pharmacologic therapy for the treatment of patients with HRS. Norepinephrine and vasopressin are acceptable alternatives in countries where terlipressin is not yet available. For patients with Type II HRS, midodrine plus octreotide appears to be an effective pharmacologic regimen that can be administered outside of an intensive care unit setting. Regardless of chosen vasoconstrictor therapy, careful monitoring is needed to ensure tissue ischemia and severe adverse effects do not occur. Artificial hepatic support devices, renal replacement therapy, and transjugular intrahepatic portosystemic shunt (TIPS) are non-pharmacologic options for patients with HRS. However, hepatic support devices and renal replacement therapies have not yet demonstrated improved outcomes and TIPS is difficult to be employed in patients with Type I HRS due to contraindications in the majority of patients. Despite advances in our understanding of hepatorenal syndrome, the disease is still associated with significant morbidity, mortality, and costs. More evidence is urgently needed to help improve patient outcomes in this difficult-to-treat population.

## 1. Introduction

Hepatorenal syndrome (HRS) is the most significant hepatorenal disorder affecting patients with advanced cirrhosis. If not treated, patients with HRS Type I survive only a median of 2 weeks and 95% of patients die within the first 30 days after onset. The median survival time is 4 to 6 months in patients with Type II HRS [1]. Patients with advanced cirrhosis and ascites are at high risk for HRS, with 18% of patients developing hepatorenal syndrome (HRS) within 1 year and up to 39% developing HRS by 5 years [2–4].

**Figure 1.**
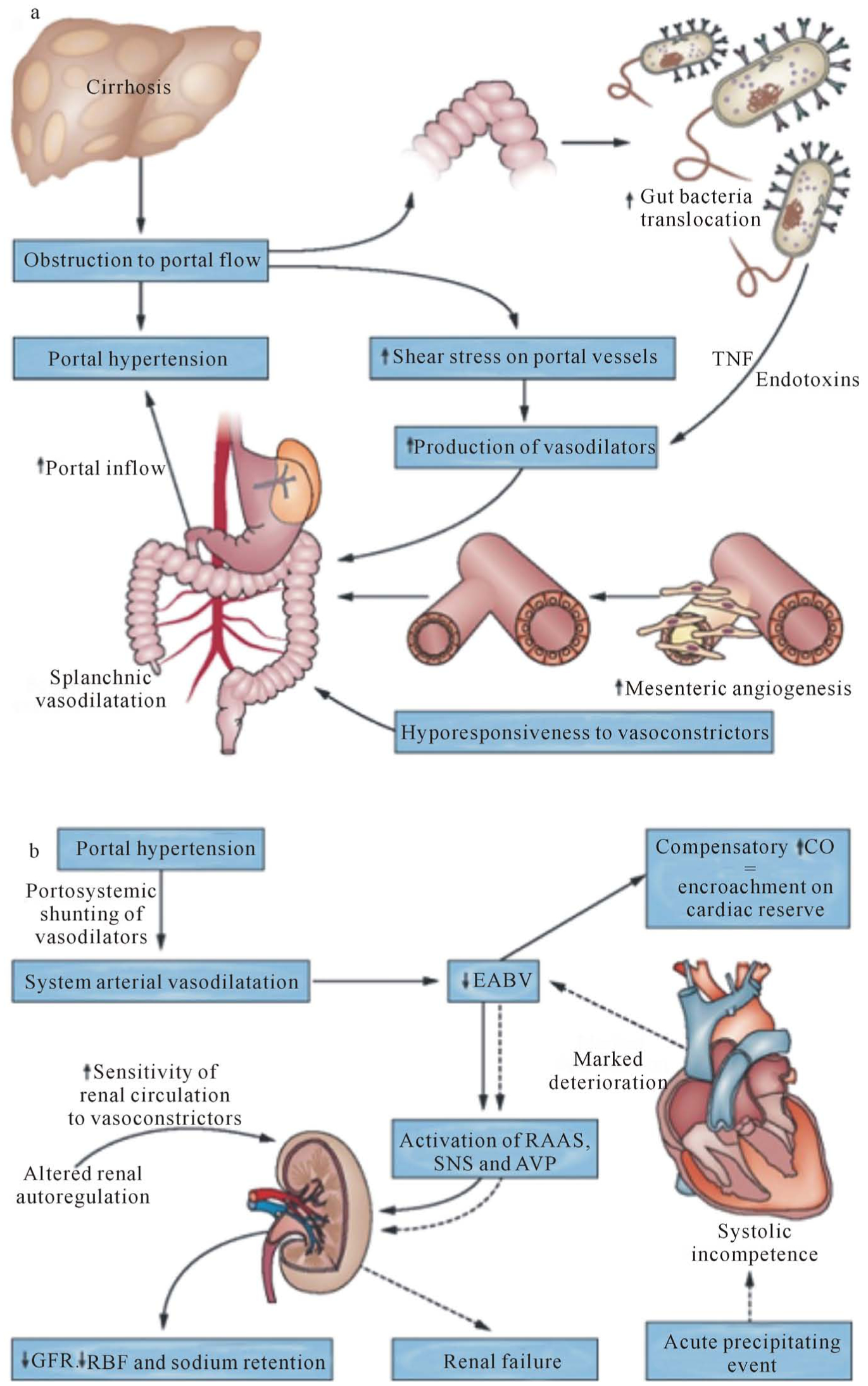
Pathophysiology of hepatorenal syndrome. a: the development of splanchnic vasodilatation and b: the development of renal dysfunction. The solid arrows indicate a baseline condition, whereas the dotted arrows indicate hepatorenal syndrome occurring in the event of a precipitating factor. Abbreviations: AVP, arginine vasopressin; CO, cardiac output; EABV, effective arterial blood volume; GFR, glomerular filtration rate; Na, sodium; RAAS, renin-angiotensin-aldosterone system; RBF, renal blood flow; SNS, sympathetic nervous system; TNF, tumor necrosis factor. *Reprinted with permission* [6].

HRS is a unique renal dysfunction, because it is a functional renal failure that occurs in the absence of parenchymal kidney disease. Patients with cirrhosis have resistance to portal flow leading to changes in shear stress of the portal vessel wall. This resultant portal hypertension is the initiating factor for HRS resulting in the production of various vasodilators including nitric oxide, carbon monoxide, cysteinyl leukotrienes, thromboxane A2, endothelin-1, and others. As portal venous pressure is increased, shear stress on the splanchic vasculature results in further release of vasodilators, causing development of splanchnic vasodilation and porto-systemic shunts reducing effective arterial blood volume and mean arterial pressure. Activation of several compensatory mechanisms, including the sympathetic nervous system, the renin-angiotensin-aldosterone system (RAAS), and the release of arginine vasopressin occurs to counteract the systemic vasodilation and increase sodium and water retention to increase intravascular blood volume. Cardiac output is increased in response to reduced effective arterial volume resulting in tachycardia and low systemic blood pressure. Ultimately, this cascade of events causes a shift in the renal autoregulation curve, making renal perfusion much more sensitive to changes in mean arterial pressure. As cirrhosis progresses, further renal vasoconstriction and sodium retention occurs as the splanchnic and systemic vasodilation worsens leading to the development of a functional renal failure (Figure 1) [5,6].

Frequently, the development of HRS is precipitated by an acute event. In patients with cirrhosis and ascites, bacterial infections appear to be the most important risk factor for the development of HRS. Infection results in circulatory dysfunction by releasing various cytokines, such as tumor necrosis factor and interleukin 6 in the splanchnic vasculature [5,6]. These inflammatory cytokines activate endothelial and inducible nitric oxide synthases increasing the production of nitric oxide [6]. As many 33% of patients who develop spontaneous bacterial peritonitis will develop HRS [7]. Other HRS triggering events include severe alcoholic hepatitis, hypovolemia as the result of excess diuresis or gastrointestinal losses (bleeding or diarrhea), large volume shifts between intravascular and extravascular compartments, and use of medications that affect afferent or efferent arteriole constriction or vasodilation in the kidney (e.g., nonsteroidal anti-inflammatory drugs [NSAIDs], angiotensin-converting enzyme inhibitors [ACE-Is], angiotensin II receptor blockers [ARBs]) [5]. Additionally, cirrhotic cardiomyopathy resulting in the inability to maintain the required cardiac reserve may be an important contributor to HRS.

## 2. Diagnosis of HRS

The assessment of HRS is daunting, because of the difficulties in providing a definitive diagnosis and the poor overall response rate to currently available therapies. The diagnosis of HRS is made by excluding all other possible causes of renal failure and utilizing revised criteria published by the International Ascites Club in 2007 (Table 1) [8,9]. These diagnostic criteria have been widely accepted; however, they may be difficult to apply in the acute care setting. The major struggle within the diagnostic criteria is the difficulty in fulfilling all of the diagnostic criteria in patients who have a presentation suggestive of HRS. One of the major limitations in the clinical setting is the ability to rule out renal failure caused by other factors, because many patients with HRS physiology have bacterial infections with or without shock, are receiving diuretic therapy prior to their AKI, or may be receiving medications or undergoing procedures that are detrimental to renal blood flow or kidney function. Additionally, patients with prolonged Type I HRS may eventually develop acute tubular necrosis due to intense renal arteriole vasoconstriction. As a result of these limitations, several renal biomarkers are being studied to help decipher HRS from other causes of AKI in patients with cirrhosis; but further studies are required before they can be applied in clinical practice [10–12].

After establishing the diagnosis, HRS can be divided into one of two forms: Type I and Type II. Type I HRS is diagnosed when there is a doubling in SCr to a value >2.5 mg/dL in a period of less than 2 weeks. Type I HRS usually develops as a result of a triggering factor that causes acute deterioration of hepatic function together with other organ dysfunctions. The most common triggers for Type I HRS are bacterial infections and severe alcoholic hepatitis. In contrast, Type II HRS occurs in patients with refractory ascites and involves renal dysfunction (SCr > 1.5 mg/dL) that is more slowly progressive and does not meet the criteria for Type I HRS. It is common for patients with Type II HRS to eventually develop Type I HRS as the result of a precipitating event [4,12].

## 3. Prevention of HRS

Prevention of Type I HRS involves appropriate identification and management of potential HRS precipitating events; whereas, prevention of Type II HRS commonly involves management of refractory ascites. In general, avoiding relative renal hypoperfusion is the key strategy for preventing HRS development. Avoiding hypovolemia by appropriately managing outpatient diuretic therapy and the discontinuation of diuretics at the first indication of AKI is extremely important. Fluid management is of critical importance and assessment of effective intravascular volume and renal perfusion pressure should be considered in hospitalized patients at risk for HRS. Fluid overload, as the result of excessive intravenous fluid administration, should also be avoided because it can be equally detrimental, resulting in hyponatremia, increased ascites, and edema. When utilizing large-volume paracentesis for the management of ascites, administration of intravenous albumin 20% to 25% (at least 6 - 8 grams per liter of ascites removed) is necessary to avoid large volume shifts from the intravascular space [13,14]. Additionally, antibiotic prophylaxis in patients with a gastrointestinal bleed can reduce the incidence of spontaneous bacterial peritonitis and renal failure and pentoxifylline therapy may reduce the incidence of HRS in patients with acute alcoholic hepatitis [15,16].

## 4. Treatment of HRS

Liver transplantation is the optimal and definitive therapy for patients with HRS because it cures the underlying organ dysfunction responsible for the pathophysiologic pathway to HRS [17–19]. Liver transplantation drastically improves mortality for patients with HRS, resulting in 5-year survival rates similar to patients without HRS who underwent liver transplantation (67.1% versus 70.1%, respectively; *P* = NS) [18]. Renal dysfunction can often be reversible and, therefore, patients with HRS are not frequently listed for combined liver-kidney transplants (LKTx); however, recommendations have been made to consider LKTx in HRS patients who have received hemodialysis (HD) for >8 weeks [14], with some groups advocating for a requirement of >12 weeks of HD prior to transplantation before consideration of LKTx [12].

Treatment of HRS with the intent to improve renal function and prolong survival long enough to allow for liver transplantation is the ultimate goal of pharmacologic therapy for HRS. Vasoconstrictors are the mainstay of therapy for HRS due to their ability to improve the hemodynamic instability that is responsible for the decreased renal perfusion pressure. The addition of albumin therapy to vasoconstrictors may further improve renal blood flow, glomerular filtration, and ultimately response rates to vasoconstrictor therapy [20]. If albumin is utilized, the admixture should provide a high concentration of albumin and the typical dose is 1 g/kg (up to 100 g) on day 1 or 2, then 25 to 50 g/day of 25% albumin (or 20 to 40 g/day of 20% albumin) thereafter [4]. Albumin therapy is continued, along with vasoconstrictor therapy, until a complete response in SCr is realized or until futility of therapy is determined. The dose and duration of albumin therapy should be dictated by volume status; as albumin is initially effective at improving intravascular volume, but will eventually result in third space volume expansion. Volume status should be assessed by hemodynamic monitoring, although the optimal method for evaluating volume status is controversial and likely includes the interpretation of several possible measurements, including heart rate, mean arterial pressure (MAP), central venous pressure, pulse pressure variation, stroke volume variation, echocardiography, urine output, ascites, and edema.

Terlipressin is a unique vasopressin analogue with potential advantages that make it the preferred vasoconstrictor for patients with HRS. The effects of vasopressin and vasopressin analogues on the V1 receptor are the predominate mechanism for treating the underlying splanchnic vasodilation present in those with HRS. There are a large number of V1 receptors in the splanchnic vasculature, making this area especially sensitive to the vasoconstrictive effects [21]. Vasoconstriction of the splanchnic vascular beds is believed to reverse HRS by increasing effective arterial blood volume, thereby suppressing activation of the renin-angiotensin-aldosterone system (RAAS) and the sympathetic nervous system, reversing compensatory renal vasoconstriction and ultimately increasing renal perfusion. Terlipressin reduces portal vein pressure and increases MAP in patients with cirrhosis and splanchnic vasodilation. It improves splanchnic blood flow and down-regulates the excessive salt and water retention that leads to ascitic fluid accumulation [22]. These attributes have made terlipressin one of the most widely used agents for the treatment of Type I HRS outside of North America.

Although the number of prospective controlled trials is small, terlipressin has been one of the most studied vasopressor agents for the treatment of HRS (Table 2) [22]. A comprehensive review of the terlipressin literature for HRS through January 2012 can be found in the Cochrane Database [23]. Combined analysis of 6 prospective studies demonstrates that terlipressin treatment improves renal function and mortality for patients with HRS. HRS reversal (reversal or complete response is defined as a decrease in SCr to a value ≤1.5 mg/dL) occurs in 25% to 50% of patients treated with terlipressin. Relapse rates after stopping therapy do occur and retreatment with vasoconstrictor therapy may be necessary. Adverse effects related to terlipressin include tachycardia, arrhythmias, chest pain, diarrhea, abdominal pain, bronchospasm, and peripheral ischemia [24]. Serious ischemic adverse events have required discontinuation of terlipressin therapy in a small percentage of patients (e.g., nonfatal myocardial infarction, livedo reticularis, and cyanosis of the fingers) [25]. The phase III Multi-Center Randomized, Placebo-Controlled, Double-Blind Study to Confirm the Reversal of Hepatorenal Syndrome Type 1 With Lucassin (Terlipressin) (REVERSE) trial [Clinicaltrials.gov identifier NCT01143246] with provide further evidence evaluating terlipressin therapy for the management of patients with HRS.

Terlipressin dosing has ranged from 0.5 to 2 mg intravenously every 4 to 12 hrs. Continuous infusion terlipressin has also been utilized, but it does not appear to offer an efficacy or safety advantage over intermittent therapy and is less convenient. Terlipressin should be initiated at 0.5 mg every 4 to 6 hours. Stepwise titration in dose (e.g., 0.5 mg increments) should be done every 1 to 2 days as tolerated if the urine output (UOP) has not improved and the SCr has not decreased from baseline (Table 3) [4]. It may take 2 to 3 days for a response in SCr to be observed, so early dosing titration decisions should focus on achieving a MAP increase of 10 mm Hg, UOP improvement, and avoidance of ischemic adverse effects. Therapy should be discontinued if patients demonstrate no response in SCr by day 4 of therapy, despite adequate titration and an increase in MAP, because a response to therapy at this point is unlikely.

In countries where terlipressin is not commercially available, other vasoconstrictor treatment options (e.g., vasopressin or norepinephrine) must still be considered. Vasopressin is considered a reasonable alternative to terlipressin therapy because of its effects on the V1 receptor; however, it is less selective than terlipressin and must be administered by a continuous infusion because of its shorter half-life. Evidence for vasopressin use in HRS comes from a retrospective study that evaluated 43 patients who had received vasopressin and/or octreotide for treatment of HRS. Response in SCr (SCR < 1.5 mg/dL) occured in 41% of the patients that received vasopressin therapy. Therapy with vasopressin, either alone or in combination with octreotide, was an independent predictor of renal function recovery (odds ratio [OR] 6.4; 95% confidence interval [CI] 1.3 - 31.8). The mean vasopressin dose in patients that responded to therapy was 0.23 ± 0.19 units/min, which is significantly higher than typically utilized in shock syndromes [26]. Although patients with cirrhosis and HRS appear to be more tolerant to higher doses of vasopressin, caution and careful monitoring of serum lactate levels and the monitoring of extremities for ischemia should be maintained for patients receiving vasopressin doses >0.1 units/min as adverse effects related to vasopressin are ischemic in nature and dose dependent [27].

Norepinephrine’s alpha-adrenergic agonist activity makes it a potent vasoconstrictor of both the venous and arterial vasculature. Similar to terlipressin, in patients with HRS, norepinephrine effectively improves UOP, sodium excretion, serum sodium concentration, creatinine clearance (CrCl), MAP, plasma renin activity, and aldosterone activity. In small comparative studies, norepinephrine has demonstrated a similar rate of HRS reversal and patient survival when compared with terlipressin [28,29]. Adverse effects between norepinephrine and terlipressin are similar, with reversible cardiac and digital ischemia being the most common adverse events [29]. The cost of norepinephrine therapy is also significantly lower than terlipressin (107 ± 31 versus 1536 ± 40 Euros, *P* < 0.0001), making it an attractive alternative therapy [28]. However, the possibility of utilizing terlipressin in patients outside of a monitored hospital setting may reduce the overall difference in cost between treatment options.

Midodrine is frequently utilized for the treatment of Type II HRS patients in North America, because it is an orally administered alpha-adrenergic agonist medication that can be administered outside of the intensive care unit. When used in combination with octreotide ± albumin it may improve length of survival and transplantation rates, particularly for patients with Type II HRS [30]. The usual midodrine dosage range for the treatment of HRS is 5 to 15 mg orally TID. If goal MAP cannot be achieved and there is no response in UOP or SCr, despite titration to 15 mg orally TID, consideration of switching to a more potent intravenous vasoconstrictor may be necessary [4].

TIPS procedures can significantly decrease the portosystemic pressure gradient. This leads to decreased plasma renin and sympathetic activity potentially improving or reversing HRS physiology [31]. Insertion of the shunt within 4 to 6 weeks of HRS onset may improve renal function recovery and survival [32]. Improvement in renal function after TIPS may take several weeks, so the use of other therapies for treating HRS is commonly required until the effect of TIPS placement is realized. TIPS can be beneficial for patients with both Type I and Type II HRS; however, many patients with Type I HRS cannot safely undergo the procedure due to their advanced liver disease or other contraindications. Patients with lower bilirubin and those with Type II HRS are more likely to have prolonged survival post TIPS [32].

Artificial liver support therapies have been evaluated for the treatment of HRS; including molecular adsorbent recirculating system (MARS), Prometheus, single pass albumin dialysis (SPAD), and single pass albumin extended dialysis (SPAED) [33–37]. These extracorporeal systems provide combined hepatic and renal support by removing water-soluble and albumin-bound toxins resulting in improved serum bilirubin, creatinine, and other laboratory measurements. Unfortunately, these artificial support systems do not provide sustained responses in kidney function after discontinuation and laboratory values commonly return to pretreatment levels after discontinuation [33]. In addition to the inability to produce meaningful outcomes, other challenges to artificial liver support systems include hypotension, blood loss each time the circuit is replaced, and the frequent need for anticoagulant administration into the extracorporeal circuit to prevent clotting of the circuit. Therefore, use of these systems for the management of patients with HRS are not recommended at this time [4,6].

Few studies have evaluated renal replacement therapy (RRT) for the treatment of HRS [38,39]. The use of RRT can improve short-term survival for patients with HRS and may be helpful with bridging patients to transplant or treating patients who have an acute reversible cause of hepatic decompensation. Use by patients who are not transplant candidates and those without an acute reversible component is unlikely to change a patient’s disease course and merely results in resource overutilization and substantial costs to the health care system [39]. Therefore, the initiation of continuous or intermittent RRT for patients with HRS is generally reserved until a significant indication for dialysis arises (e.g., severe hyperkalemia, metabolic acidosis, or volume overload). Individual patient selection, according to the severity of illness, Child Pugh and MELD scores, and the potential for liver transplantation should all be considered prior to the initiation of RRT.

## 5. Conclusions

HRS is the most significant disease within the spectrum of hepatorenal disorders and is associated with a substantial mortality rate. HRS physiology is characterized by splanchnic arterial vasodilation causing reduced effective arterial volume, renal vasoconstriction as a result of activation of the sympathetic nervous system and the RAAS, reduced cardiac output as the result of cirrhotic cardiomyopathy, and release of vasoactive mediators that affect renal blood flow and glomerular microcirculatory hemodynamics. Patients with Type 1 HRS have a more acute rise in their SCr values and shorter survival times as compared to patients with Type II HRS.

Early identification and management of HRS is critical to the success of the chosen treatment. The only definitive treatment established for HRS is liver transplantation.

The goal of all other pharmacologic and nonpharmacologic therapies is to prolong survival time enough to allow for liver transplantation. Several small studies have attempted to evaluate various pharmacologic agents for the treatment of HRS, but only vasoconstrictors combined with albumin therapy have emerged as a preferred initial treatment option. Terlipressin, in combination with albumin volume expansion, is the preferred pharmacologic therapy for the management of patients with HRS. Norepinephrine and vasopressin are alternatives if terlipressin is unavailable. Midodrine with octreotide appears to be an effective pharmacologic regimen in patients with Type II HRS and those who require an alternative to intravenous therapy. Although TIPS is effective for both Type I and II HRS, it is less commonly employed in Type I HRS patients due to the presence of contraindications to the procedure. Artificial hepatic support devices and renal replacement therapy are effective for correcting abnormal laboratory values, but have not demonstrated the ability to reverse HRS. The decision to deliver these therapies should be limited to patients who have an indication for dialysis and are high on the liver transplant list.

Substantial advances in understanding the pathophysiology and management of HRS has improved the recognition and treatment of HRS patients. However, several questions still remain regarding how best to optimize current treatment options. Given the substantial morbidity, mortality, and cost associated with HRS management; more studies are urgently needed to help improve patient outcomes in this difficult population.

## Figures and Tables

**Table 1.** Diagnostic criteria for hepatorenal syndrome.

Diagnostic Criteria for Hepatorenal Syndrome
Cirrhosis with ascites
Serum creatinine (SCr) >1.5 mg/dL (>133 umol/L)
No improvement in serum creatinine levels (decrease to of ≤1.5 mg/dL) after at least 2 days with diuretic withdrawal (if on diuretics) and volume expansion with 20% to 25% albumin. The recommended dose of albumin is 1 g/kg of body weight per day, up to a maximum of 100 g/day.
Absence of shock
No current or recent treatment with nephrotoxic medications
Absence of parenchymal kidney disease as defined by proteinuria <500 mg/day, no microhematuria (<50 red blood cells per high power field), and normal renal ultrasonography
**Classification of Hepatorenal Syndrome**
Type I — doubling in SCr to a value > 2.5 mg/dL in a period of less than 2 weeks
Type II — stable or more slowly progressive renal dysfunction (SCr > 1.5 mg/dL) not meeting the criteria for Type I HRS

Adapted from Solerno, *et al*. [9] and Gines, *et al*. [5].

**Table 2.** Selected clinical studies of vasoconstrictors in the treatment of HRS.

Study Design	No. of Patients	Therapy	Significant Outcomes
Prospective, Randomized [40]	N = 46 (35 Type I and 11 Type II HRS)	Terlipressin + Albumin versus Albumin	Renal function improvement more likely in terlipressin + albumin (43.5% versus 8.7%, *P* = 0.017)
Prospective, observational[20]	N = 21 (16 Type I and 5 Type II HRS)	Terlipressin + Albumin versus Terlipressin	Albumin administration found to predict renal function response (77% responders versus 25% responders, *P* = 0.03)
Prospective, randomized [41]	N = 24 (Type I HRS)	Terlipressin versus Placebo	Terlipressin significantly improved UOP, CrCl, MAP, and decreased SCr compared with placebo. At day 15, 5 of 12 patients receiving terlipressin survived compared with 0 of 12 patients receiving placebo (*P* < 0.05).
International, Multi-center, Randomized [25]	N = 112 (Type I HRS)	Terlipressin versus Placebo	Treatment success: terlipressin 25% versus placebo 12.5%, *P* = 0.093. HRS reversal: terlipressin 34% versus 13%, *P* = 0.008. Related adverse effects: terlipressin 9% versus placebo 2%, *P* = NS
Prospective, randomized [24]	N = 52 (Type I HRS)	Terlipressin + Albumin versus Albumin	80% complete response with terlipressin + albumin versus 19% response with albumin (p < 0.01). Improved survival at 180 days with terlipressin + albumin (p < 0.01)
Retrospective [26]	N = 43 (32 Type I and 11 Type II HRS)	Vasopressin + Octreotide versus Vasopressin versus Octreotide	Complete response higher in patients receiving vasopressin or vasopressin + octreotide versus octreotide monotherapy (*P* = 0.01)
Prospective, open label [29]	N = 40 (Type I HRS)	Norepinephrine versus Terlipressin	Reversal of HRS occurred in 50% of patients in each treatment group (p = NS). Survival was similar between groups (p = 0.8). Baseline creatinine clearance, MAP, and plasma renin activity were independent predictors of response.

UOP = urine output; CrCl = creatinine clearance; MAP = mean arterial pressure; SCr = serum creatinine; *Adapted from Kiser et al*. [22].

**Table 3.** Dosage and administration of vasoconstrictor medications for HRS.

Vasoconstrictor Agents	Dosing Recommendations
Terlipressin	0.5 to 2 mg IV q4 to 6 hours; increase dose by 0.5 mg increments every 1 to 2 days if there is no improvement in SCr as long as no side effects are present. Goal MAP increase of 10 mm Hg from baseline. Maximum dose = 12 mg/day.
Vasopressin	0.01 to 0.8 units/min continuous IV infusion. Increase dose by 0.05 units/min every 30 to 60 minutes to achieve a 10 mm Hg increase in MAP from baseline or a MAP > 70 mm Hg
Norepinephrine	0.05 to 1 mcg/kg/min (5 to 75 mcg/min) continuous IV infusion. Titrate every 30 minutes to achieve a 10 mm Hg increase in MAP from baseline
Midodrine + Octreotide	Midodrine 5 to 15 mg PO TID. Titrate to achieve a 10 to 15 mm Hg increase in MAP from baseline. Octreotide: 100 to 200 mcg SQ/IV TID; or 25 to 50 mcg IV bolus, followed by 25 to 50 mcg/hour continuous infusion (no titration)

SCr = serum creatinine; MAP = mean arterial pressure; IV = intravenous; 1) Adjunctive albumin administration is recommended: 1 g/kg (up to 100 g) on day 1 or 2, then 25 to 50 g/day of 25% albumin (or 20 to 40 g/day of 20% albumin) thereafter. 2) Therapy should be discontinued after 4 days if no response in SCr is observed, despite adequate dosage titration, because the likelihood of a response to therapy is low. 3) All patients should be monitored for signs of ischemia (*i.e*., visual evaluation of digits, distal pulses, abdominal pain, serum lactate, and/or troponin) at least every 12 hours and after any dosing titration. 4) In patients that demonstrate a complete response to therapy, dosage reduction or vasoconstrictor discontinuation should be attempted by day 14 of therapy to determine the sustainability of the response. Restarting therapy may be necessary if a relapse occurs; *Adapted from Nadim et al*. [4]
